# Can an online clinical data management service help in improving data collection and data quality in a developing country setting?

**DOI:** 10.1186/1745-6215-12-190

**Published:** 2011-08-08

**Authors:** Maarten A Wildeman, Jeroen Zandbergen, Andrew Vincent, Camelia Herdini, Jaap M Middeldorp, Renske Fles, Otilia Dalesio, Emile van der Donk, I Bing Tan

**Affiliations:** 1 Department of Head and Neck Oncology and Surgery, The Netherlands Cancer Institute- Antoni van Leeuwenhoek Hospital, Amsterdam, The Netherlands; 2 Department of Biometrics, The Netherlands Cancer Institute- Antoni van Leeuwenhoek Hospital, Amsterdam, The Netherlands; 3 Department of Otorhinolaryngology, Gadjah Mada University, Yogyakarta, Indonesia; 4 Department of Pathology, VU University Medical Centre, Amsterdam, the Netherlands; 5 Department of Otorhinolaryngology, Academic Medical Center (AMC), Amsterdam, The Netherlands

## Abstract

**Background**

Data collection by Electronic Medical Record (EMR) systems have been proven to be helpful in data collection for scientific research and in improving healthcare. For a multi-centre trial in Indonesia and the Netherlands a web based system was selected to enable all participating centres to easily access data. This study assesses whether the introduction of a Clinical Trial Data Management service (CTDMS) composed of electronic Case Report Forms (eCRF) can result in effective data collection and treatment monitoring.

**Methods**

Data items entered were checked for inconsistencies automatically when submitted online. The data were divided into primary and secondary data items. We analysed both the total number of errors and the change in error rate, for both Primary and Secondary items, over the first five month of the trial.

**Results**

In the first five months 51 patients were entered. The Primary data error rate was 1.6%, whilst that for Secondary data was 2.7% against acceptable error rates for analysis of 1% and 2.5% respectively.

**Conclusion**

The presented analysis shows that after five months since the introduction of the CTDMS the Primary and Secondary data error rates reflect acceptable levels of data quality. Furthermore, these error rates were decreasing over time. The digital nature of the CTDMS, as well as the online availability of that data, gives fast and easy insight in adherence to treatment protocols. As such, the CTDMS can serve as a tool to train and educate medical doctors and can improve treatment protocols.

## Background

Data collection concerning medical needs is required to assess the effectiveness of interventions and current health care practices [1]. Furthermore, data collection by Electronic Medical Record (EMR) systems has been proven to be helpful in data collection for scientific research and can be helpful in improving healthcare. These EMR systems allow for the early identification of missing data and the patients possibly loss-to-follow-up, which is essential for the conduct of proper scientific research [2-6].

A Clinical Trial Data Management service (CTDMS) has been introduced for running a multicenter clinical trial in Indonesia and in the Netherlands. The same system has also been introduced for monitoring treatment results of Nasopharyngeal Carcinoma (NPC) in Indonesia.

In most countries NPC is an orphan disease, but overall has a worldwide incidence of 80.0000 new cases per year, being endemic in Northern Africa, Southern China and Hong Kong, and the South-East Asian peninsula, including Malaysia, Vietnam, Thailand, Singapore and Indonesia.

In Indonesia NPC is the most frequent cancer in the head and neck area and ranks as the 4^th ^most common tumour found in males. The incidence is estimated 6 per 100.000, leading to 12.000 new cases per year [7,8]. Little is known about treatment results of NPC in Indonesia.

The CTDMS system was selected because of the web-based nature which makes the data approachable for all participating parties. This online accessible data system has made it easier for the principal investigator to check the data for inconsistencies. The senior physician can easily see if treatment is according protocol.

This study assesses whether the introduction of CTDMS composed of online Case Report Forms (eCRF) can result in improved patient outcomes. The assessment focuses on data quality and the identification of possible bottle necks within the patient care process.

This study investigates if a web based CTDMS can be helpful in proper data collection by analysing errors in data items. Bottle necks in patient care are analysed by comparison of treatment plan and actual treatment.

## Methods

The CTDMS is constructed for the NPC Clinical Trial: Early detection of primary and recurrent NPC using (anti-)EBV based tumour markers and evaluation of primary treatment for NPC (funding KWF NKI-2008-4233). A technical description of the CTDMS is provided in Appendix 1. The database is comprised of 10 online eCRF's. In order to prevent errors from being entered, data validation rules were implemented into the eCRF's prior to commencement of the NPC Clinical trial. These data validation rules assess whether certain pre-specified conditions are valid and can therefore pin-point omissions or erroneous data. Online warning messages notify the data-manager (entering data) when errors are detected. Commonly used checks are, for instance, range checks that verify whether values are within the boundaries dictated by the study protocol, and mandatory field checks (i.e. 'This field cannot be blank').

Of the 10 eCRF's, 9 were required to be completed multiple times per patient during the study and only 1 was to be completed and submitted once per patient. Each of these submissions is a unique realization of the form. For example, for one patient a laboratory form is completed during baseline measurements, just before the start of the treatment. Once this form is submitted through the CTDMS, there is one realization of the laboratory form stored in the database for this patient. After the patient received treatment, a laboratory form is completed and submitted again. The data base then contains two realizations of the laboratory form, for that patient. Each realization may be submitted multiple times if it contained errors. We note that it is impossible to claim that an entered form for which no warning messages were displayed is clean, as new errors may be found later.

The data-manager completing an eCRF has the option to ignore (override) a warning message, however in such cases, he/she is required to provide an explanation which is recorded in an Audit Trail entry field (error log). Warning messages and error logs are also created when an incorrect value or data-type is entered, an omission is detected, or when a previously entered value is changed. Changed values are considered to be (previously undetected) errors that have now been rectified (except when the changed value also triggers a check to fire, in which case the data is considered unclean).

The eCRF's contain differing quantities of data. Each field to be entered is considered a data item, which were designated either as primary or secondary. Primary data items are data that were considered essential for the assessment of the NPC Clinical trial primary endpoint, and so for assessment of treatment protocol. Secondary data items are data required to assess the clinical trial's secondary endpoints.

As acceptable levels of data quality, an 1% error rate for primary and an 2.5% error rate for secondary data points were adopted [9]. We present the change in error rate over the course of the trial, the number of errors per submission, and the change in data quality per form per submission.

## Results

Between November 2009 and March 2010 a total of 4860 data items pertaining to 51 patients were entered. This is the first five months of an estimated 3 year long accrual period. In total 433 eCRF's were submitted, of which 329 were unique realizations. Each CRF has been submitted between 1 to 4 times. Table 1 presents an overview of the submitted eCRF's and data items.

**Table 1:** Number of times each form has been entered and the number of data items per form

	Patients	Forms*	Data Items	Primary	Secondary	Total Items
Patient information/Physic	51	75	18	7	11	1350
Radiology diagnostics	49	92	13	0	13	1196
New Complaint	3	4	2	0	2	8
PDT Reaction/Adverse event	1	2	3	0	3	6
General patient information	23	34	8	4	4	272
PDT Therapy form	2	2	6	6	0	12
Endoscopy/NPC Diagnostics	40	68	6	6	0	408
Primary Tumor & NPC Diagn.	2	2	14	5	9	28
Laboratory form	48	64	5	3	2	320
Pathology, Staging & Given Treatment	48	90	14	9	5	1260

Totals:		433	89	40	49	4860

*Number of forms submitted, some unique realizations have been submitted multiple times.

**Figure 1:**
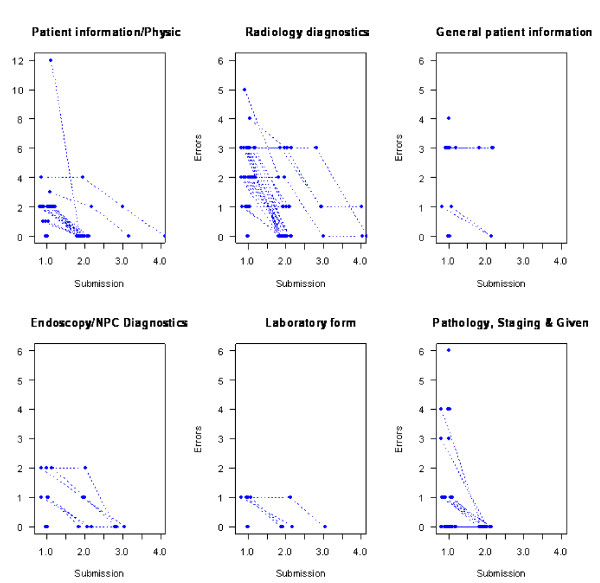
**Errors per submission for the six forms of the nine forms for which there have been more than one submission (per realization)**.

Of the 433 submitted eCRF realizations, 287 were submitted for the first time without primary data errors (Table 2), while 253 forms (realizations) were submitted for the first time without secondary data errors (Table 3). No form had more than two errors in the primary data. One form contained 10 secondary data errors when it was submitted for the first time. This was baseline patient registration data for which the wrong patient was entered. In general subsequent submissions contained fewer errors (Figure 1).

**Table 2:** The number of forms submitted with erroneous primary data items at each submission

		Number of errors		
		**0**	**1**	**2**
**Submission Number**	**1**	287	31	11
	**2**	78	9	2
	**3**	8	3	0
	**4**	3	1	0

For example 287 forms were entered error free at the first submission, while four were entered for the fourth time, 3 error free and one with one error.

**Table 3:** The number of forms submitted with erroneous secondary data items at each submission

		Number of errors					
		0	1	2	3	4	10
**Submission Number**	1	253	19	39	15	2	1
	2	76	2	8	3	0	0
	3	9	0	2	0	0	0
	4	4	0	0	0	0	0

For example, the "Pathology, Staging & Given treatment" eCRF contained a total of 89 unique data items, with the number of data items per eCRF ranging from 2 to 18 (Table 1). Of these 89 items, 40 were classified as primary data, with the remaining 49 being classified as secondary. The error rate at first submission was 3.3% for primary data and was 8.4% for secondary data.

**Figure 2:**
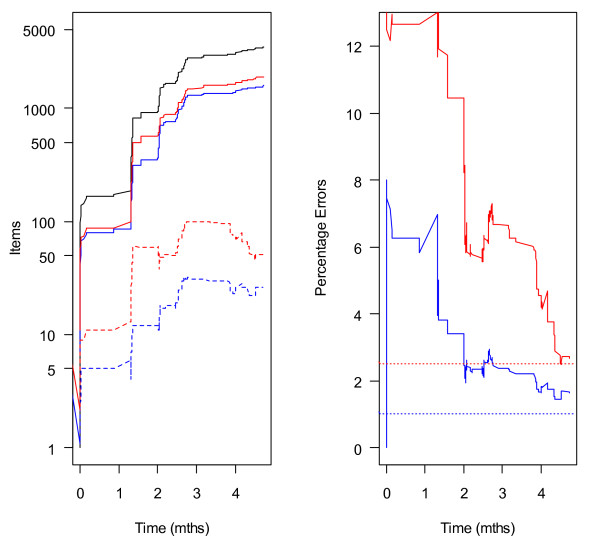
**(A) The cumulative number of unique data items entered (black), the number of primary data items entered (blue solid) and errors unresolved (blue dashed), the number of secondary data items entered (red solid) and errors outstanding (dashed lines)**. (B) The percentage of Primary (blue) and Secondary (red) errors unresolved over time. The horizontal lines are the targets for a final analysis.

To assess the change in data quality over time, the proportion of unsolved errors in primary and secondary data were plotted against time (in months). Figure 2(A) presents the cumulative number of unique data items submitted and the number of unresolved errors over the first five months of the study. Figure 2(B) presents the change in the percentage of unresolved errors of primary and secondary data items. Although the absolute number of unsolved errors is increasing with time (due to the accrual of patients), the fraction of erroneous data is declining. Five months after the start of study the error rate for the primary data items was 1.6% and for the secondary data items the error rate was 2.7%. Although not quite at the levels appropriate for final analysis, the standard of data quality is high, very early into the study.

## Discussion

For this study we found an error rate of 1.6% for primary data items, while in earlier studies in the same setting data could not be analysed because of the massive data loss and poor data quality. With this real time data monitoring and inbuilt checks we have realized acceptable levels of data quality. The CTDMS prevents us from missing data or ending up with poor quality data at the end of the study, which often at that point cannot be resolved anymore.

The presented analysis shows that after five months since the introduction of the CTDMS the error rates for both Primary and Secondary data items reflect acceptable levels of data quality. Furthermore these error rates were decreasing over time. The drop in errors per form with each form submission indicates that, while being prompted by the CTDMS, the data manager and responsible doctors are actively solving the errors. Online warning messages notify the data manager (entering data) when errors are detected, allowing them to immediately correct the data, rather than the usual delay associated with paper based CRFs.

Clearly, the CTDMS encourages local data managers to verify the entered data and, if necessary, ask the doctor whether the information is correct. It is also likely that the reason that data managers have to specify arguments before submitting the form in case the CTDMS detects erroneous data motivates them to verify whether the available data is actually correct. This may explain why our results show a significant increase of clean data and a self-learning curve of the data manager is to be expected. Moreover, the error logs provide valuable information about the bottlenecks in the treatment of the NPC patients.

In the past authors have pointed out that existing data collections in developing countries are often deficient [10,11]. Eiseman and Fossum (2005) emphasize that existing data collections are insufficiently comprehensive, sometimes inaccurate, and often out of date by the time the data can be acted upon. All point out that without these data the required empirical knowledge to address the health problems in developing countries is insufficient. Especially on strategic planning, priority setting, monitoring and evaluation, advocacy, and general policymaking [12-14].

These comments supported us on introducing an online medical record system which could play an important role in improving data collection and data quality. Accordingly, during analysis we have also seen that treatment procedures are often unsatisfactory. The first analysis regarding the treatment of NPC has been presented and discussed with all members of the disciplinary team. The main concern was the duration of the radiotherapy. According to the protocol the duration for administering the 66 gray radiotherapy should take to the utmost 42 days, yet analysis showed that the treatment time takes in average 66 days, which will lead to inadequate treatment [15,16]. Future analysis has to show if intervention by CTDMS system-based education of the doctors will eventually lead to better treatment outcome. The digital nature of the CTDMS, as well as the online availability of data, gives fast and easy insight in adherence to treatment protocols. As such, the CTDMS can serve as a tool to directly train and educate medical doctors. Therefore, a potential even bigger advantage of an online medical record system is the ability to monitor the data from the teaching hospitals especially in developing countries. Via this way the teachers can communicate directly or visit the participating hospitals with a custom fit teaching program, which will make such visits more effective.

## Conclusion

We show that an online clinical data management service can improve data quality in a developing country setting. In the future we expect to see both less loss-to-follow-up and better treatment programmes with help of this CTDMS. For better and more efficient medical care programs and studies in developing countries we believe an online data system is essential.

The digital nature of the CTDMS, as well as the online availability of that data, gives fast and easy insight in adherence to treatment protocols. As such, the CTDMS can serve as a tool to train and educate medical doctors and can improve treatment protocols. Since the introduction of this system training the doctors has become much more efficient.

## Abbreviations

EMR: Electronic Medical Record; CTDMS: Clinical Trial Data Management service; CRF: Case Report Forms; eCRF: electronic Case Report Forms; NPC: Nasopharyngeal Carcinoma; EBV: Epstein Barr Virus.

## Competing interests

Since the original submission of the article E. Van der Donk, as CEO of FormsVision BV, has gained the right to commercialize the ALEA Software. All other authors declare to have no competing interests.

## Authors' contributions

MW and JZ designed the clinical record forms, conceived the study, participated in the analyses and wrote the manuscript. AV analysed the data and helped writing the manuscript. CH introduced the system in Yogyakarta. JM, RF and OD helped designing the study and the clinical record forms. ED and OD provided the online system. ED was responsible for technical support. IT supervised the execution of this study. All authors read and approved the final manuscript.

## Appendix 1

### Technical description CTDMS

The selected CTDMS is ALEA™ 3.0, which uses Microsoft Infopath 2007 for eCRF template design and Sharepoint Enterprise 2007 for rendering the eCRF's in browsers. SQL server 2008 is used for data storage. The CTDMS provides a comprehensive eCRF. It uses standard browsers running on any computer connected to the internet. The system has been validated, and has been certified by registered auditors, as being in compliance with relevant regulations, such as the FDA's CFR 21 Part 11.

The CTDMS eCRF design module is based on an industry grade enterprise electronic forms system: Microsoft Infopath 2007 for form design and Microsoft Forms Server 2007 for data entry. The components make use of a common standard representation of data and metadata: the Operational Data Model of CDISC. Within the CTDMS, the components share a database for storing and retrieving information about the trial, and a separate database for storing and retrieving patient data.

The online Data Management Module of the CTDMS is a web browser application that supports online completion of eCRF for healthcare studies. It requires initial login with a username and password, and provides a navigation menu for all trials to which the account has been granted access, and the selected investigators for which the account has been granted permissions to access. Transmission of data is SSL encrypted using RSA 1024 bit Public Key encryption.

Data validation rules were implemented into the eCRF's using the tools Microsoft Office InfoPath provides, as well as some Xpath expressions. With data validation rules implemented, the eCRF automatically checks the data as soon as it is entered. If a value does not match the specified condition, an error alert provides the user with immediate feedback. Moreover, after completion of an eCRF, the user is prompted to provide an explanation of all data items which raised validation errors. This enables users to submit data with validation errors, while providing a comprehensive audit trail in compliance with requirements from regulatory authorities. Examples of data validation rules, which trigger error flags is provided in Table 4.

**Table 4:** List of filters/rules which trigger error flags

Name eCRF	Data item	Data validation rule
Patient information/ Physical examination form	Date of First Visit	1. The field Date of First Visit is mandatory and cannot be blank. 2. The Date of First Visit cannot be after the Date of today

	Date of Birth	1. The patient Date of Birth cannot be blank 2. The patient Date of Birth cannot be after the current date. 3. The Date of birth you entered is before 1-1-1900, the patient is too old to participate in this study

	Date of Signed informed consent	1. The field "Date of Signed informed consent" is mandatory and cannot be left blank 2. The Date of First Visit cannot be after the Date of today

		1. The field Heart Rate cannot be blank. 2. The field "heart rate" cannot be more than 220 p/m 3. The field "heart rate" cannot be less than 40 p/m

	Temperature	1. Patient Temperature should be between 35 and 41.5 degrees

	WHO performance rate"	1. The field "performance rate" is mandatory and cannot be left blank 2. During First Visit, the "WHO performance rate" cannot be either 3 or 4.

Radiology Diagnostics form	CT/MRI- scan Date Sample Taken	1. The field CT/MRI- scan Date Sample Taken is mandatory and cannot be blank. 2. The Date of sample taken, CT/MRI-scan, cannot be before the Date of Visit, as specified on the General Patient Information form (belonging to this Event/Visit). 3. The Date Sample Taken CT/MRI- scan cannot be before the Date of First Visit. 4. The Date Sample Taken CT/MRI- scan during PDT Assessment should be about 12 weeks after the Date of Foscan Administration and no less than 10 weeks after that date. 5. The Date of CT/MRI- scan cannot be later than today.

	T-stage	1. The T-stage (0,1,2A and 2B,3,4) in the field "CT-MRI T-stage" has to be the same as the T (0,1,2A and 2B,3,4) in the field "T-stage" on the Pathology form. 2. The T- stage cannot be "0" if patient has been included in this study because of a recurrent or persistent disease

	field CT/MRI Lesion Size	1. The field CT/MRI Lesion Size (length) cannot be blank when the field T-stage has been entered. 2. The field CT/MRI Lesion Size (length) cannot be more than 200 mm

	CT/MRI site	1. The field "CT/MRI site" is mandatory and cannot be left blank 2. The tumour site CT/MRI- scan selected here differs from the Suspicious site as specified on the Endoscopy form during this Visit. Are you sure this is correct? If yes, please provide explanation in Audit Trail entry after Validation 3. The tumour site CT/MRI- scan selected here differs from the Tumour site as specified on the Endoscopy form during this Visit. Are you sure this is correct? If yes, please provide explanation in Audit Trail entry after Validation

	endoscopy	1. If Nose endoscopy has been performed, the field Date of Endoscopy cannot be blank 2. During (visit) Positive Test, the Date of Endoscopy should be after the Date of Endoscopy of the PDT Assessment. 3. The Date of Endoscopy should be after the Date of Endoscopy of the last PDT Follow Up 4. The Date of Endoscopy should be after the Date of Endoscopy of the PDT Visit(s) 5. The Date of Endoscopy should be after the Date of Endoscopy of the previous Follow Up visit. 6. The Date of Endoscopy should be after the Date of Endoscopy of the First Visit. 7. The Date of Endoscopy should be 3 months after the Date of Endoscopy of the Therapy Assessment

Examples of data validation rules triggering error flags for a subset of the data items on two of the eCRF's.

## References

[B1] Pollak VE Lorch JA Effect of electronic patient record use on mortality in End Stage Renal Disease, a model chronic disease: retrospective analysis of 9 years of prospectively collected data BMC Med Inform Decis Mak 2007 7 38 10.1186/1472-6947-7-38 18045495 PMC2238736

[B2] Fraser HS Jazayeri D Nevil P Karacaoglu Y Farmer PE Lyon E An information system and medical record to support HIV treatment in rural Haiti BMJ 2004 329 1142 1146 10.1136/bmj.329.7475.1142 15539669 PMC527691

[B3] Simbini T Computerised information management systems in HIV/AIDS care and outcomes research Cent Afr J Med 2006 52 65 67 18254459

[B4] Clifford GD Blaya JA Hall-Clifford R Fraser HS Medical information systems: a foundation for healthcare technologies in developing countries Biomed Eng Online 2008 7 18 10.1186/1475-925X-7-18 18547411 PMC2447839

[B5] Choi SS Jazayeri DG Mitnick CD Chalco K Bayona J Fraser HS Implementation and initial evaluation of a Web-based nurse order entry system for multidrug-resistant tuberculosis patients in Peru Stud Health Technol Inform 2004 107 202 206 15360803

[B6] Fraser H Choi SS Galipot M Jazayeri D Mangubat N Successful transfer of a Web-based TB medical record from Peru to the Philippines AMIA Annu Symp Proc 2006 924 PMC1839702 17238543

[B7] Devi BC Tang TS Corbex M Reducing by half the percentage of late-stage presentation for breast and cervix cancer over 4 years: a pilot study of clinical downstaging in Sarawak, Malaysia Annals of oncology: official journal of the European Society for Medical Oncology/ESMO 2007 18 1172 1176 10.1093/annonc/mdm105 17434897

[B8] Curado MP Edwards B Shin HR Cancer Incidence in Five Continents, Vol. IX. 2007 IARC Scientific Publications No. 160, Lyon, IARC 2007 Ref Type: Data File

[B9] Sullivan EMGMASRCCG A Statistically-based process for Auditing Clinical data listings Drug Information Journal 1997 31 647 653

[B10] Eiseman E Fossum D The Challenges of Creating a Global Health Resource Tracking System RAND Corperation 2005

[B11] Heathfield H Pitty D Hanka R Evaluating information technology in health care: barriers and challenges BMJ 1998 316 1959 1961 9641938 10.1136/bmj.316.7149.1959 PMC1113407

[B12] Clifford GD Blaya JA Hall-Clifford R Fraser HS Medical information systems: a foundation for healthcare technologies in developing countries Biomed Eng Online 2008 7 18 10.1186/1475-925X-7-18 18547411 PMC2447839

[B13] Mantas J Ammenwerth E Demiris G Hasman A Haux R Hersh W Recommendations of the International Medical Informatics Association (IMIA) on Education in Biomedical and Health Informatics. First Revision Methods Inf Med 2010 49 105 120 10.3414/ME5119 20054502

[B14] McKee M Foege W, Black R, Daulaire N A decade of experience in eastern Europe Leadership and management for improving global health 2005 167 186

[B15] Fu KK Pajak TF Trotti A Jones CU Spencer SA Phillips TL A Radiation Therapy Oncology Group (RTOG) phase III randomized study to compare hyperfractionation and two variants of accelerated fractionation to standard fractionation radiotherapy for head and neck squamous cell carcinomas: first report of RTOG 9003 Int J Radiat Oncol Biol Phys 2000 48 7 16 10.1016/S0360-3016(00)00663-5 10924966

[B16] Thames HD Kuban D Levy LB Horwitz EM Kupelian P Martinez A The role of overall treatment time in the outcome of radiotherapy of prostate cancer: an analysis of biochemical failure in 4839 men treated between 1987 and 1995 Radiother Oncol 2010 96 6 12 10.1016/j.radonc.2010.03.020 20400191

